# Decreased Triple Network Connectivity in Patients with Recent Onset Post-Traumatic Stress Disorder after a Single Prolonged Trauma Exposure

**DOI:** 10.1038/s41598-017-12964-6

**Published:** 2017-10-03

**Authors:** Yang Liu, Liang Li, Baojuan Li, Na Feng, Lihong Li, Xi Zhang, Hongbing Lu, Hong Yin

**Affiliations:** 1 School of Biomedical Engineering, Fourth Military Medical University, Xi’an, Shaanxi China; 2 Department of Physiology, Fourth Military Medical University, Xi’an, Shaanxi China; 3 Department of Engineering Science and Physics, The City University of New York at College of Staten Island, Staten Island, New York, USA; 4 Department of Radiology, Xijing Hospital, Xi’an, Shaanxi China

## Abstract

The triple network model provides a common framework for understanding affective and neurocognitive dysfunctions across multiple disorders, including central executive network (CEN), default mode network (DMN), and salience network (SN). Considering the effect of traumatic experience on post-traumatic stress disorder (PTSD), this study aims to explore the alteration of triple network connectivity in a specific PTSD induced by a single prolonged trauma exposure. With an arterial spin labeling sequence, three networks were first identified using independent component analysis among 10 PTSD patients and 10 healthy survivors, who experienced the same coal mining flood disaster. Then, the triple network connectivity was analyzed and compared between PTSD and non-PTSD groups. In PTSD patients, decreased connectivity was identified in left middle frontal gyrus of CEN, left precuneus and bilateral superior frontal gyrus of DMN, and right anterior insula of SN. The decreased connectivity in left middle frontal gyrus of CEN was associated with clinical severity. Furthermore, no significant connection of SN with CEN and DMN was found in PTSD patients. The decreased triple network connectivity was found in this study, which not only supports the triple network model, but also suggests a possible neurobiological mechanism for cognitive dysfunction of this type of PTSD.

## Introduction

Post-traumatic stress disorder (PTSD) is an anxiety disorder that develops after exposure to a terrifying event or ordeal in which grave physical harm occurred or personal security was threatened^1^. Neuroimaging studies have identified a number of structural and functional alterations in some brain regions associated with PTSD, e.g., hippocampus^2,3^, amygdala^4,5^, anterior cingulate gyrus, cingulate cortex^6^, and medial prefrontal cortex^5^. Instead of focusing on specific brain regions, it is now increasingly recognized that the analysis of brain networks can help to explain the complex neurobiological mechanisms underlying psychiatric disorders^7,8^. Thus, a series of networks have been identified by characterizing a set of functionally connected brain regions (nodes) with temporally correlated patterns, called intrinsic connectivity networks (ICNs). In psychopathology, Menon^7^ proposed a triple network model, which assumed that the dysfunction of three key ICNs may play an important role in affective and neurocognitive symptoms across several psychiatric disorders, e.g., schizophrenia, depression, and Alzheimer’s disease. The three key ICNs involved in the triple network model include the central executive network (CEN), default mode network (DMN), and salience network (SN). It has proposed that aberrant intrinsic connectivities within and between CEN, DMN, and SN are characteristics of many psychiatric and neurological disorders^7^. As a typical psychiatric disorder, it would help us understand the neurobiological mechanism underlying PTSD through the possible alteration of its triple network connectivity (within and between three key ICNs).

Currently, increasing evidence has proved the dysfunction within CEN^9^, DMN^10^, and SN^11^ in PTSD. Among them, three studies observed distinct connectivity alterations within the three ICNs of PTSD patients^8,12,13^. Based on functional magnetic resonance imaging (fMRI) studies of the survivors experiencing the Wenchuan earthquake, connectivity variations within three ICNs were identified in both adult^12^ and pediatric PTSD patients^13^, though the changed patterns seem not identical. In addition, decreased connectivity within the three ICNs was identified in PTSD patients experiencing multiple incidents of childhood sexual and/or physical abuse^8^. These studies indicate that though aberrant connectivity within three ICNs of the triple network model has been identified, the observations seem inconsistent, which may be partly due to different types of traumatic experiences^14,15^. In addition, there is no study investigating the connectivity between these three ICNs of PTSD patients.

In contrast to most PTSD studies focusing on subjects who experienced repeated and short-duration traumas^1^, such as earthquake or abuse experience, few studies have investigated the effect of recent onset PTSD induced by a single prolonged trauma exposure. In our previous studies, which used structural and perfusion MRI data acquired from survivors experiencing a mining flood disaster, a series of structural and functional alterations were identified at nodes of the three ICNs, including volume deficits in left hippocampus and parahippocampal gyrus^3^, cortical thinning in the left precuneus and right parahippocampal gyrus^1^, and cerebral blood flow (CBF) deficits in bilateral frontal lobes and right superior frontal gyrus^16^. The integration of these findings suggests possibly decreased connectivity within these ICNs, due to volume, cortical thickness, and CBF deficits.

To investigate possible alteration of the triple network connectivity in patients with PTSD induced by a single prolonged trauma exposure, in this study, further analysis was performed between survivors with PTSD and without PTSD, who experienced the same coal mining flood disaster. Based on CBF maps estimated from pulsed arterial spin labeling (ASL) time series^16^, we first extracted independent components (ICs) from CBF time-series to identify three ICNs. Then the reconstructed network connectivity within and between the three networks were compared between two groups. Finally, the relationship between connectivity changes and clinical symptoms was investigated.

## Results

### Identification of ICs based on the templates

**Figure 1:**
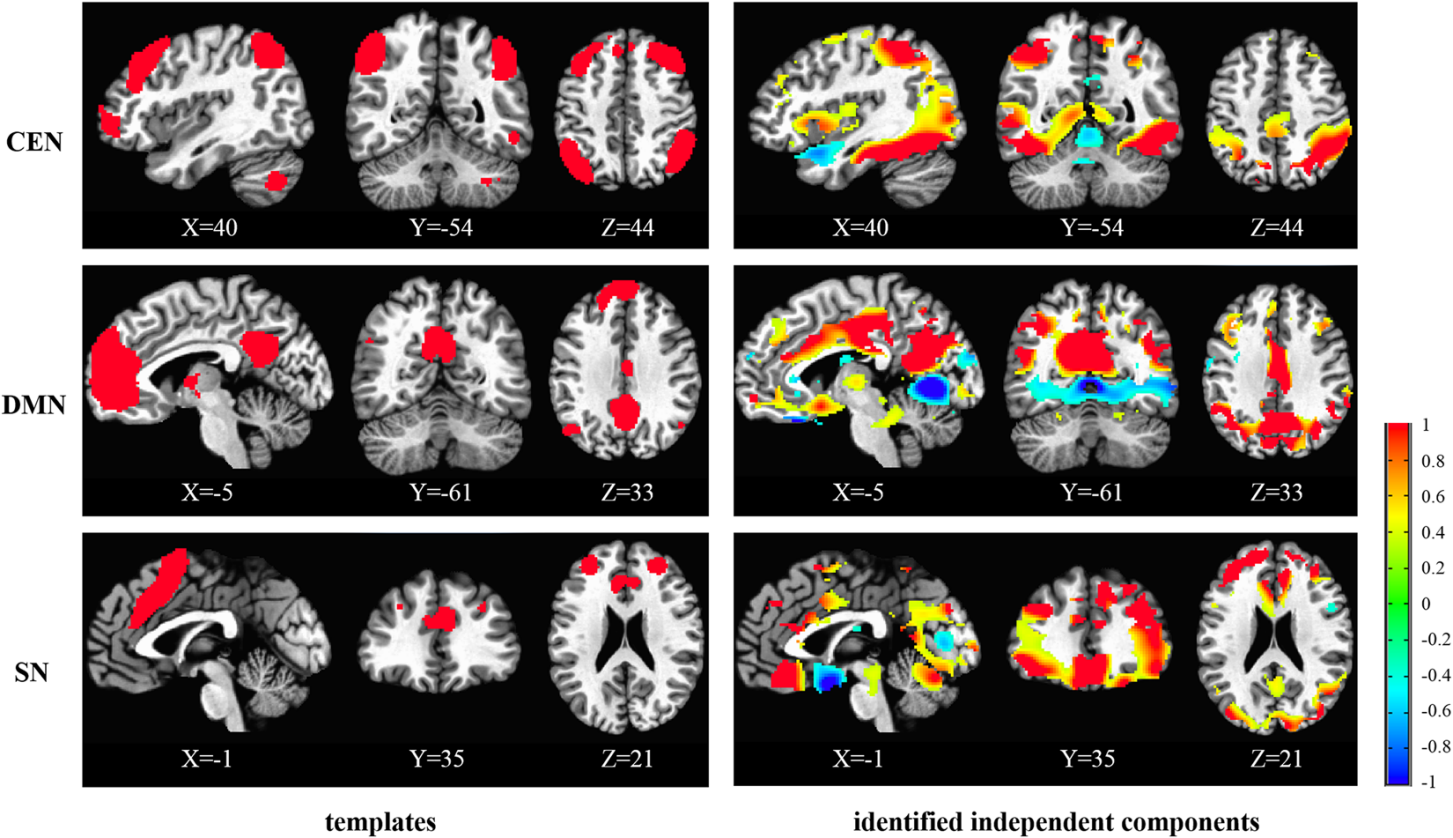
The templates and identified independent components of CEN, DMN, and SN. The color bar indicates the Z values of templates and independent components. The MNI coordinate is indicated under each template and corresponding independent component.

After decomposition, 20 spatial ICs can be obtained. Based on the spatial correlation between ICs and templates of CEN, DMN, and SN, the central executive, default mode, and salience components were identified (Fig. 1). The spatial correlations between the templates of CEN, DMN, and SN and the identified ICs are 0.065, 0.098, 0.023, respectively. Seen from Fig. 1, the most major regions in the templates were also included in the identified ICs.

### Decreased CEN connectivity in patients with PTSD

**Figure 2:**
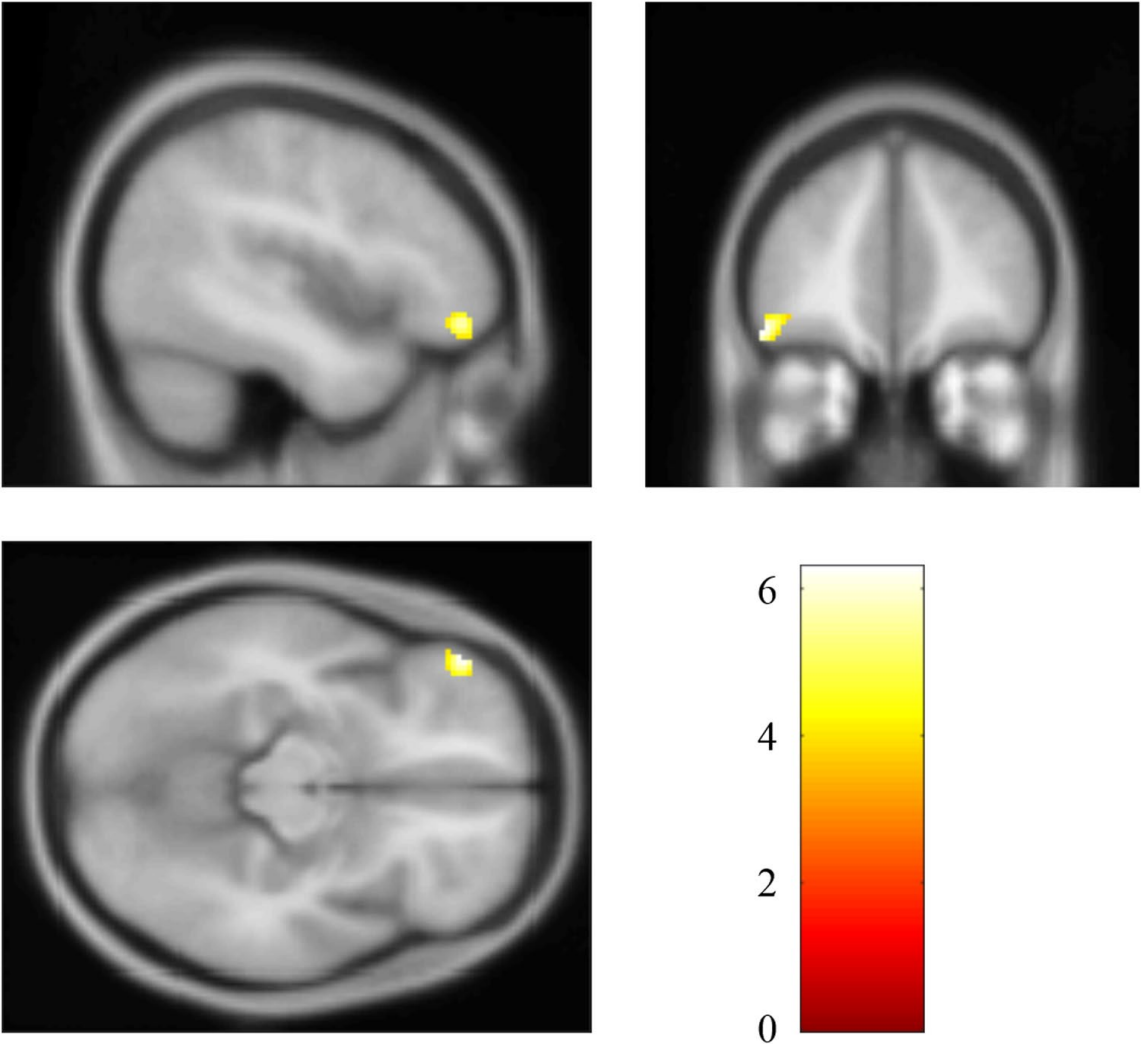
Brain region with significant decreased CEN connectivity in PTSD patients compared with non-PTSD survivors.

**Figure 3:**
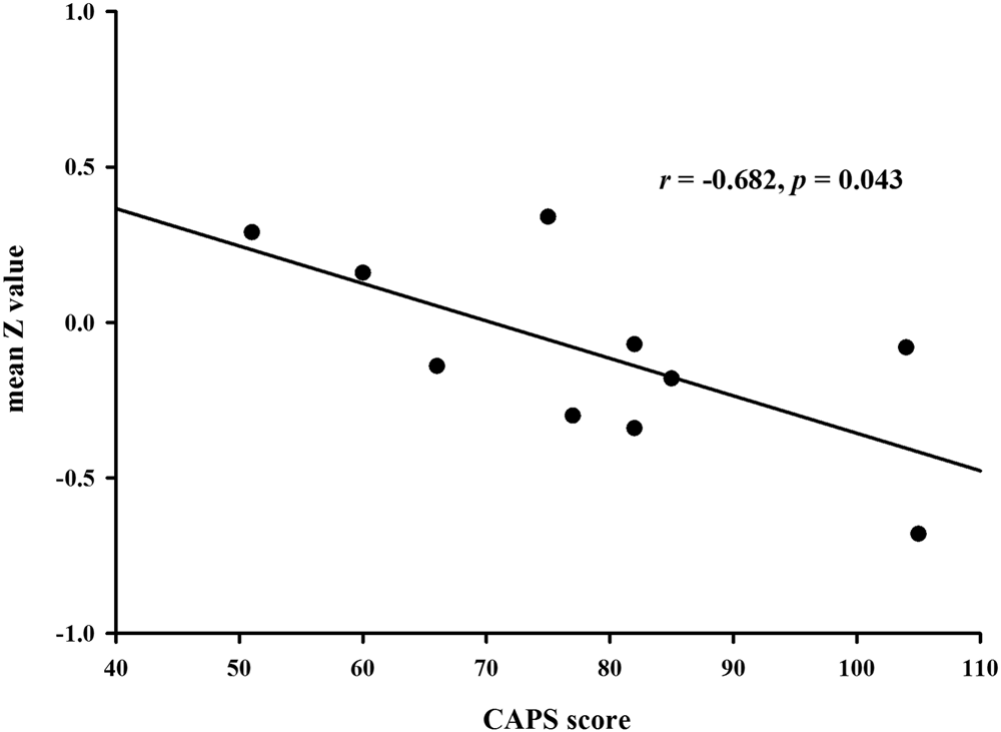
Mean Z value of the left middle frontal gyrus correlated negatively with the CAPS score of PTSD patients.

The whole-brain analysis revealed significantly decreased CEN connectivity in the left middle frontal gyrus (MNI: −48, 44, −14; cluster size: 77; *t* score: 6.2846), compared with the non-PTSD group (Fig. 2). The post hoc analysis found that mean connectivity strength (Z value) of the left middle frontal gyrus in CEN was correlated inversely with the symptom severity of the disorder, Clinician-Administered PTSD Scale (CAPS) scores, in PTSD patients (*r* = −0.682, *p* = 0.043), as shown in Fig. 3.

### Decreased DMN connectivity in patients with PTSD

**Figure 4:**
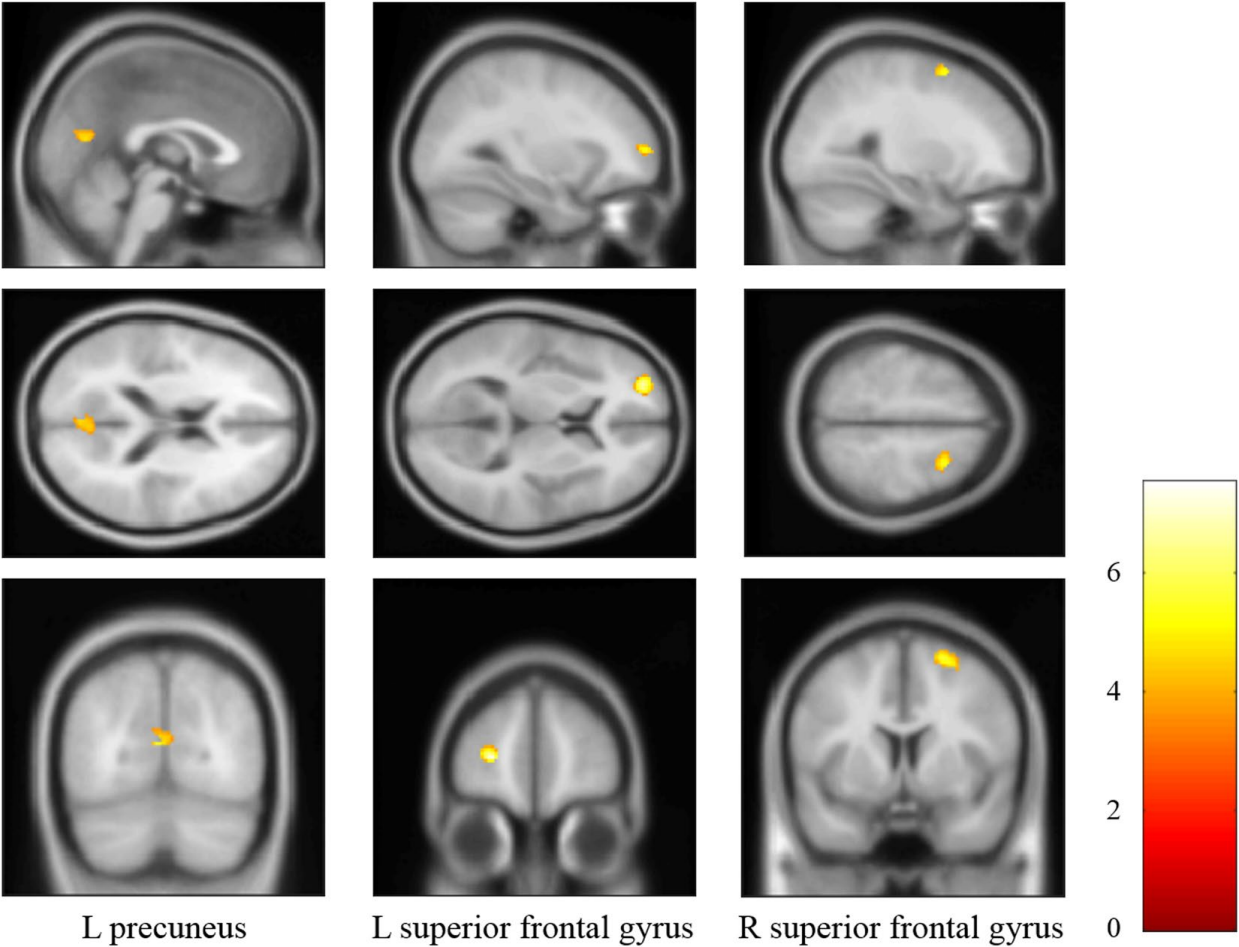
Brain regions with significant decreased DMN connectivity in PTSD patients compared with non-PTSD survivors.

The whole-brain analysis revealed significantly decreased DMN connectivity in the left precuneus and bilateral superior frontal gyrus of PTSD patients, compared with the non-PTSD group (Table 1, Fig. 4). The correlation analysis indicated that there was no significant correlation between mean Z values in above regions and the CAPS scores.

**Table 1:** Regions with significantly decreased DMN connectivity in PTSD group compared with non-PTSD group.

Brain regions	MNI (X, Y, Z)	Cluster size	*t* score
L precuneus	−4, −72, 14	78	5.3736
L superior frontal gyrus	−26, 56, 6	52	7.5462
R superior frontal gyrus	22, 8, 62	47	5.9519

### Decreased SN connectivity in patients with PTSD

**Figure 5:**
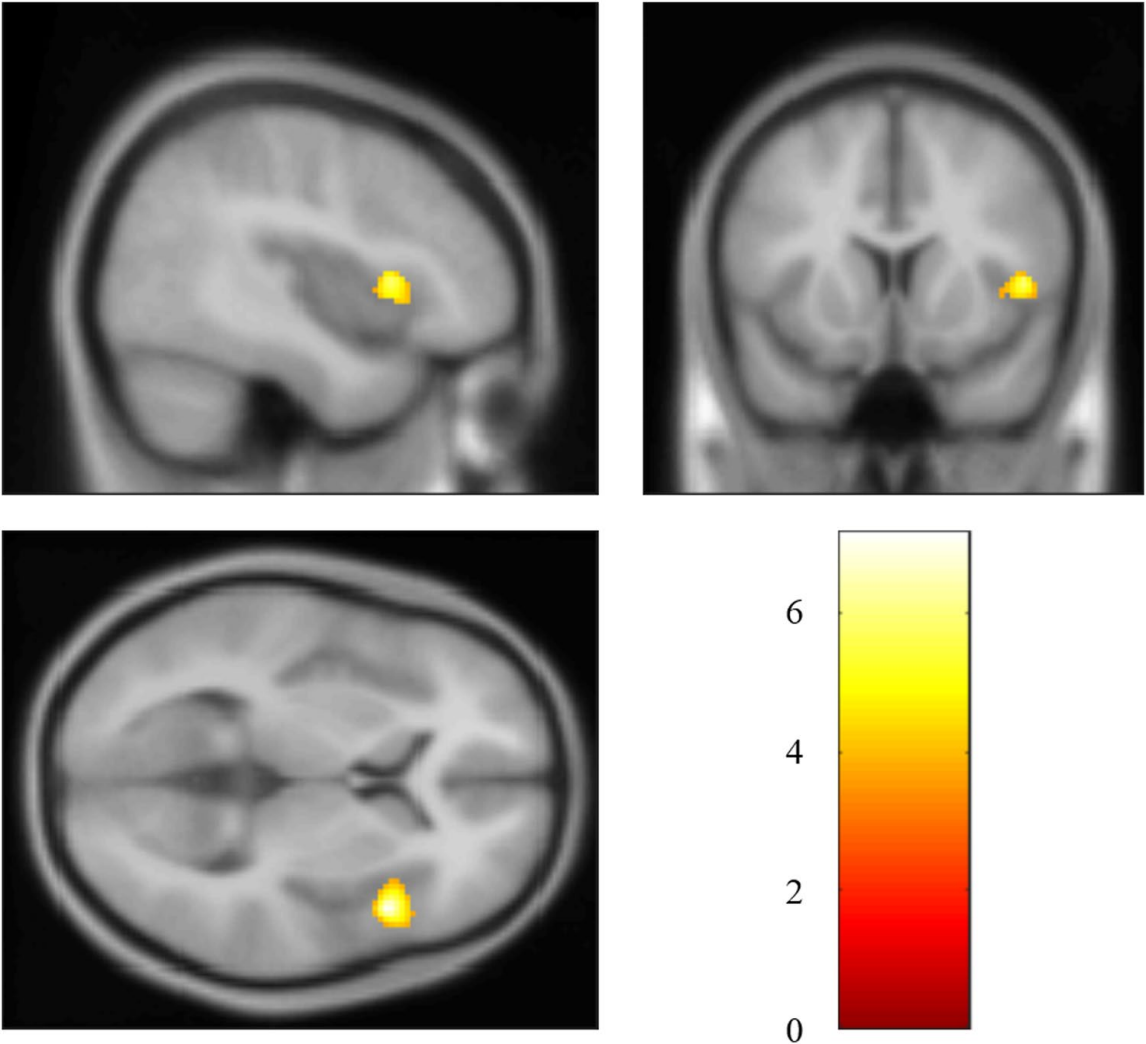
Brain regions with significant decreased SN connectivity in PTSD patients compared with non-PTSD survivors.

The whole-brain analysis revealed significantly decreased SN connectivity in the right anterior insula (MNI: 46, 14, 6; cluster size: 74; *t* score: 7.1769), compared with the non-PTSD group (Fig. 5). No significant correlation between mean Z value of this region and the CAPS score was found.

### Functional network connectivity (FNC) analysis between CEN, DMN, and SN

Table 2 reports the significant pair-wise correlations between CEN, DMN, and SN in PTSD and non-PTSD groups, respectively, and the correlation differences between two groups. The results of one-sample *t* test showed that significant pair-wise correlations between CEN, DMN, and SN were found in non-PTSD group, while only significant correlation between CEN and DMN was found in PTSD group. The results of two-sample *t* test indicated that no significant FNC difference was found between PTSD and non-PTSD groups.

**Table 2:** Pair-wise correlation coefficients (*R*) between CEN, DMN, and SN in PTSD and non-PTSD groups, and two-sample *t* test between two groups, respectively, assessed by statistical analysis on network connectivity.

ICN pair	PTSD (one-sample *t* test)		non-PTSD (one-sample *t* test)		PTSD vs. non-PTSD (two-sample *t* test)
	*R*	*p*	*R*	*p*	
CEN-DMN	0.43	<0.005	0.45	<0.005	NS
CEN-SN	0.13	NS	0.14	<0.005	NS
DMN-SN	0.11	NS	0.2	<0.005	NS

NS: not significant with *p* < 0.05.

## Classification Performance

**Figure 6:**
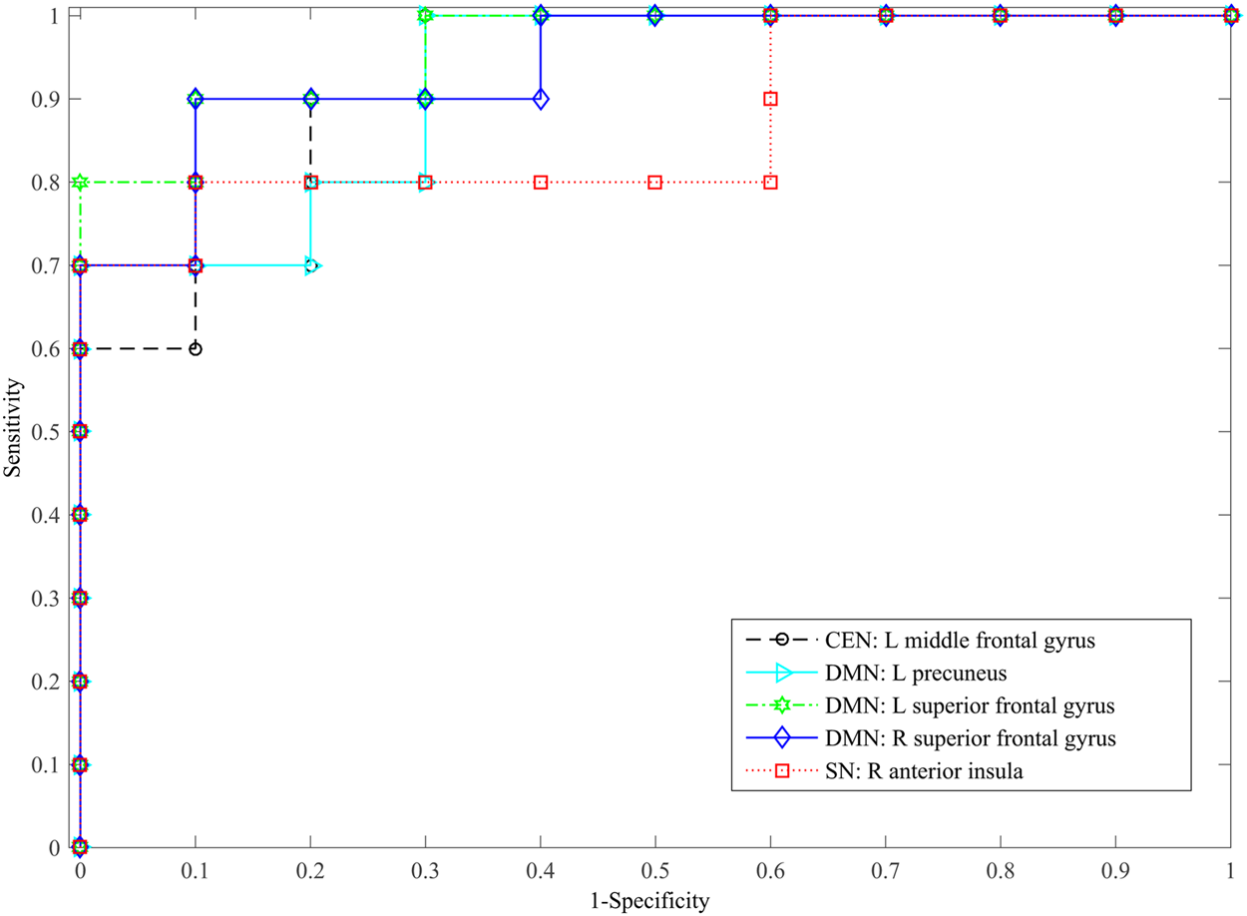
ROC curves of each identified region with the SVM classifier.

Considering the limited sample size, a cross validation was performed to evaluate above identified results if they have significant differences in within- and between-network connectivity. With the cross validation, the performance of each identified region within three ICNs in differentiating PTSD from non-PTSD groups was shown in Table 3. It indicates that the classification accuracies of these regions are all greater than or equal to 0.85. The receiver operating characteristics (ROC) curves of each identified region with the support-vector machine (SVM) classifier were also plotted, as shown in Fig. 6. Since no significant FNC difference between three ICNs was found between PTSD and non-PTSD groups, we did not further evaluate this result.

**Table 3:** The classification performance using the identified regions within three ICNs.

Brain regions	Sensitivity	Specificity	Accuracy	AUC
CEN: L middle frontal gyrus	1	0.7	0.85	0.92
DMN: L precuneus	0.7	1	0.85	0.92
DMN: L superior frontal gyrus	0.9	0.9	0.9	0.96
DMN: R superior frontal gyrus	0.9	0.8	0.85	0.94
SN: R anterior insula	0.8	0.9	0.85	0.87

AUC: area under the curve of the receiver operating characteristics.

## Discussion

The present study investigated alteration of the triple network connectivity between survivors with and without recent onset PTSD from a coal mining flood disaster, using the pulsed ASL sequence. Based on our analysis results, the assumption of decreased connectivity within three key ICNs has been verified in the left middle frontal gyrus of CEN, left precuneus and bilateral superior frontal gyrus of DMN, and right anterior insula of SN. Meanwhile, the decreased connectivity in left middle frontal gyrus of CEN was identified to be associated with the clinical severity (CAPS score). Though no significant alteration of network connectivity between three key ICNs was found between the PTSD and non-PTSD groups, the connectivity patterns of two groups seem somehow different (non-PTSD: significant pair-wise connections were found between all three ICNs; PTSD: significant connection was found only between CEN and DMN). These results indicated that the decreased triple network connectivity may contribute to cognitive dysfunction in this specific type of PTSD (recent onset PTSD induced by a single prolonged trauma exposure).

After decomposition of CBF series and identification of networks by the spatial correlation with templates, we obtained the components corresponding to the CEN, DMN, and SN. In this study, the spatial correlations between the templates of CEN, DMN, and SN and the identified ICs were 0.065, 0.098, and 0.023, respectively. Compared to typical correlation range reported in blood oxygen level-dependent (BOLD) fMRI studies, the correlation values reported in this study are relatively low. This may be due to the templates used, which are designed for BOLD data. Different from relatively consistent distribution of BOLD signals in the whole brain, the ASL signal of gray matter is triple the value of white matter. After the decomposition with ICA, this difference may result in relatively low correlations between the templates and ICs. Please note that though the correlations were low, the most major regions of three networks were effectively identified by corresponding ICs, as shown in Fig. 1, demonstrating the reasonability of selected components. Nevertheless, these identified components may contain some other regions, while some other ICs may contain some regions of the three ICNs (CEN, DMN, and SN). In this study, by using the identified components, only regions with significantly different connectivity between the PTSD and non-PTSD groups, within these three ICNs, were reported. In addition, to evaluate the reliability of the identified regions with decreased connectivity, a leave one out cross validation was performed by using the identified regions to differentiate the PTSD group from non-PTSD subjects. The validation results (Table 3 and Fig. 6) further confirm the reliability of reported findings.

The CEN is responsible for high-level cognitive functions, e.g., planning, control of attention, and working memory^11^, which mainly includes the dorsal lateral prefrontal cortex (DLPFC) and the posterior parietal cortex^17^. The middle frontal gyrus identified in this study is the key region of DLPFC^18^, which is important for the execution of emotion regulation strategies^19^. It is reported that decreased activity in middle frontal gyrus was identified in PTSD patients when reappraising negative pictures^9^ and increased activity in this region was found after successful psychotherapy^20^. Thus, the decreased connectivity of left middle frontal gyrus may indicate impaired ability of emotion regulation in PTSD patients. Moreover, it was further identified that the decreased connectivity in this region associated with clinical severity, suggesting a putative biomarker and a possible target for the improvement of emotion regulation for this type of PTSD.

The DMN has been recognized to play a key role in self-referential processing, autobiographical memory, and cognition^7^. The alterations in the DMN may be a potential mechanism underlying depersonalization and related identity disturbances that follow PTSD^11^. The DMN involves posterior cingulate cortex, precuneus, inferior parietal gyrus, angular gyrus, middle temporal gyrus, superior frontal gyrus, and medial frontal gyrus^17^. In this study, decreased connectivities in left precuneus and bilateral superior frontal gyrus of DMN were identified in PTSD patients. The precuneus is regarded as the central hub of the DMN^21^ and associates with source memory processing^22^. Especially, the left precuneus is related to mental imagery and posterior buffer working memory^23^. The connectivity level of the precuneus has been proved to be negatively correlated to anxiety and depression symptomatology^24^. The superior frontal gyrus plays a key role in attention shifting^25^ and its activity increases linearly with the need for executive control^26^. Therefore, the decreased connectivity in DMN may indicate its weakened role in coordinating with CEN to control emotion and concentration in PTSD patients. Together with decreased connectivity in CEN mentioned above, it suggests that decreased connectivity in and between CEN and DMN may partially contribute to the psychopathology disorder of PTSD.

The SN is located at the interface of the cognitive, homeostatic, motivational, and affective systems of the human brain, suggesting its key role in integrating cognition, action, and feelings^27^. Decreased insula activation is regarded to underlie over- and under-modulation of emotion^28^ and alterations in interoceptive awareness of PTSD^11^. Crucially, altered connectivity in SN may be related to attentional capture enhanced by error signals^29^, resulting in hypervigilance and hyperarousal symptoms in PTSD^11^. It indicates that the increased anterior insula activity after effective treatment would help to restore the integrity of SN and reestablish emotional awareness, thereby alleviating the hyperarousal symptoms^11^. With anterior insula as the dynamic hub, SN is deemed to mediate the engagement of the CEN and disengagement of the DMN^11^, thereby contributing to a variety of complex brain functions through dynamic interplay between attention and cognitive-affective processing. In addition, the insula of SN plays a critical role in switching between the CEN and DMN^30^. The decreased connectivity in SN may indicate less coordination of the two networks. Putting all the consideration together, the decreased connectivity of anterior insula identified in SN may suggest under-modulation of SN within and between CEN and DMN in the PTSD patients.

The above hypothesis may be reflected by decreased pair-wise connectivity between SN, CEN, and DMN. To investigate possible connection alterations between the three networks, FNC analysis was performed and the results indicated that no significant alteration of network connectivity was found between PTSD and non-PTSD groups, which may be due to small sample size used in this study. However, compared with significant connections between any two of the three ICNs in non-PTSD group, no significant connection between SN and CEN or DMN was found in PTSD patients, suggesting a weaken coordinating role of SN in the triple network model. With further consideration of decreased regional connections within CEN, DMN, and SN, our study has found decreased triple network connectivity in PTSD patients who experienced a single prolonged trauma exposure, which may shed some light on neurobiological mechanism of cognitive dysfunction for this type of PTSD.

The present study was an extension of our previous structural and functional studies using the same subjects, in which we have identified a series of nodes in three key ICNs with volume deficits^3^, cortical thinning^1^, and CBF deficits^16^. Combined with decreased triple network connectivity presented in this study, our multimodality studies indicate that structural and functional deficits may contribute to cognitive dysfunction of the specific PTSD. These deficit regions and loosely connected ICNs identified in the triple network may be further treated as putative biomarkers and therapeutic targets. The work presented here also supports the importance of the triple network model in understanding dysfunctions in cognitive disorders.

Several limitations of this study should be addressed. Firstly, the sample size is small. For subjects surviving from a coal mining flood disaster, we could not control the sample size, just like some studies on PTSD induced by other sudden disasters, such as a fire disaster^31^ or sarin attacks^4^. Based on the given sample size, we’ve tried our best to make all data consistent to alleviate possible sampling bias. Secondly, the results could not survive after multiple comparison correction. That may be due to the small sample size used in this study and the subtle alteration of functional connectivity caused by early stage of PTSD. Considering the relatively lower *p* value of 0.001 for connectivity analysis, relatively high accuracy of the cross validation, and consistent identification of decreased triple network connectivity with our previous structural and functional findings, it’s believed that the finding in decreased triple network connectivity in this study is relatively generalized. Thirdly, all the participants were trauma-exposed and no normal subjects without PTSD were included. It is reported that trauma exposure, rather than PTSD, disrupts the connectivity of networks^32^. Further studies are underway to investigate the effects of trauma exposure with the addition of a non-exposed control group.

## Materials and Methods

### Subjects

All of the twenty subjects were right-hand males that survived from a coal mining flood disaster, in the Henan province of China. In the mining disaster, 69 miners were trapped for 72 hours, and all of them were rescued and survived. After that, 48 survivors were hospitalized and received a medical checkup, excluded from any cerebrovascular disorders. Six months later, 17 survivors of them met the diagnostic criteria for PTSD, and 10 agreed to participate in this MRI study as the PTSD group. The PTSD was diagnosed using DSM-IV^33^ and the Structured Clinical Interview for DSM-IV^34^. The severity of their symptoms was assessed using the Chinese version of the CAPS^35^. Meanwhile, 10 out of 31 survivors without PTSD agreed to participate in the MRI study as the non-PTSD group. The elapsed time between the traumatic event and MRI scans ranged from 187 to 190 days.

With the recruited PTSD and non-PTSD groups in this study, each group had 10 male subjects. As expected, the PTSD group had higher CAPS scores than those of non-PTSD group (PTSD: 78.72 ± 17.2, non-PTSD: 31.40 ± 18.57, *p* < 0.001) and two groups significantly differed in age (PTSD: 40.80 ± 6.83, non-PTSD: 34.30 ± 5.37, *p* = 0.032)^16^. The study was conducted according to the principles in the Declaration of Helsinki and approved by the Institutional Board of the Fourth Military Medical University. All subjects received a comprehensive description of the MRI study and gave voluntarily written informed consent before entering the study.

### Data acquisition

All MRI scans were acquired on a 3.0T MR scanner (MAGNETOM Trio, Siemens AG, Erlangen, Germany). ASL perfusion images were acquired using a pulsed ASL sequence, with imaging parameters set as TR = 2700 ms, TE = 13 ms, slice thickness = 3.0 mm, acquisition matrix = 64 × 64, flip angle = 90°, field of view (FOV) = 224 × 224 mm, TI_2_ = 1800 ms, and slice = 25 with 1.5 mm gap. TI_2_ increased with 45 ms per slice. For each subject, the ASL dataset contains 121 images, including a M0 images and 60 label/control pairs. Meanwhile, T1-weighted images covering the whole-brain (176 sagittal slices) were acquired by a 3D magnetization prepared rapid acquisition gradient echo (MPRAGE) sequence, with acquisition parameters set as TR = 1900 ms, TE = 2.26 ms, TI = 900 ms, flip angle = 9°, acquisition matrix = 256 × 256, FOV = 220 × 220 mm, and 1.00 mm slice thickness with no inter-slice gap.

### Data processing

Currently, most analyses on functional connectivity have been performed by using BOLD fMRI^11,12^. Based on measuring differences in oxygen consumption, BOLD-fMRI can provide a relative measurement of blood perfusion. In contrast, ASL sequence can provide the absolute quantification of CBF^36^, which may help interpret the ambiguous results concerning functional connectivity of networks in mental diseases^36,37^. Our previous study used effective partial volume (PV) correction method to improve the quality of ASL image, which has been confirmed to be beneficial to perfusion and connectivity analysis^16^.

The image analysis was performed on a computer workstation installed with MATLAB 7.11.0, Statistical Parameter Mapping (SPM8, Wellcome Department of Imaging Neuroscience, London, UK; http://www.fil.ion.ucl.ac.uk/spm/software/spm8/), and custom-built Matlab package^16^.

For each subject, control and label image series of pulsed ASL dataset were realigned separately to correct head movement. The corresponding structural image was segmented to generate posterior probability maps of gray matter, white matter and cerebrospinal fluid, using the standard proce ssing step of SPM. Then, the segmented structural image and the mean image generated from realigned pulsed ASL sequence were co-registered to obtain the percentage of different-type tissues at each voxel of the perfusion map, based on the transformation of structural and pulsed ASL coordinates to the MNI coordinate. The differences of label/control pairs were calculated to generate a total sixty difference images.

To get accurate estimation of CBF maps, the well-established linear regression method was first utilized to correct the PV effect in ASL data induced by low spatial resolution^38^. Then the two compartment model was adopted to calculate the CBF maps^39^. In this study, a total of sixty CBF maps were acquired for each participant and constituted a CBF time series with all 3D spatial frames ordered along the time axis by the acquisition time. After that, each map of the CBF series was normalized to the MNI space and spatially smoothed with an 8-mm Gaussian kernel.

### Group ICA and identification of networks

All the preprocessed CBF series were analyzed using the group independent component analysis (ICA) in the fMRI toolbox GIFT^40^ (GIFT 4.0a, Medical Image Analysis Lab, University of New Mexico, Albuquerque, New Mexico; http://mialab.mrn.org/software/gift/index.html). When the CBF series were decomposed by the ICA algorithm, the spatially ICs and the corresponding time course of each subject were generated after the back-reconstruction step. The size of time course of each subject is *M* × *N*, where *M* representing the number of label/control pairs and *N* is the same as the number of decomposed ICs. Then the spatial ICs identified by correlation with templates were used for the analysis within three ICNs, and the time courses were used for the analysis between three ICNs.

In this study, 20 ICs were isolated using the Infomax algorithm, based on the work on ICs stability^41^. Then the CEN, DMN, and SN templates (http://findlab.stanford.edu/functional_ROIs.html) were used to identify the central executive, default mode, and salience components, respectively, based on the spatial correlation between ICs and templates^42^. Using the identified ICs of CEN, DMN, and SN, the second-level analysis was first performed. In the time course of each subject, each column corresponds to one IC. Based on the identified ICs for CEN, DMN, and SN, the corresponding columns were selected from the time course of each subject and used for FNC analysis.

### Second-level analysis

Based on the identified ICs of CEN, DMN, and SN, voxel-wise one-sample *t* tests (*p* < 0.001, uncorrected) were used to obtain the spatial pattern of each network in both groups. To take age effect into account, two-sample *t* tests (*p* < 0.001, uncorrected) were performed to compare each ICN between PTSD and non-PTSD groups with age as a covariate.

### Post hoc correlation analysis

Based on the second-level analysis, brain regions with significantly different (increased or decreased) connectivity strengths (Z values) between the PTSD and non-PTSD groups were selected as regions of interest (ROIs). To investigate the relationship between Z values and symptom severity, a post hoc Pearson’s partial correlation analysis was performed between mean Z value of each ROI and the CAPS scores of PTSD patients. Here age was also treated as a controlling covariate and the significance level was set as *p* < 0.05.

### Functional network connectivity analysis

Though the ICs are spatially independent, significant temporal correlations may exist. Such correlation can be used to characterize between-networks functional connectivity. Using the selected columns from time course of each subject, FNC analysis was performed to calculate the pair-wise connectivity between the three ICNs by the FNC toolbox (http://mialab.mrn.org/software/). In theory, FNC was performed by computing a constrained maximal lagged correlation between two columns of time course for each subject^43^, which ranges from −1~1. The maximum possible lag was set as 3 s. With this lag, the maximal correlations were computed between any two of the three ICNs (CEN, DMN, and SN). One-sample *t* test was performed for each group to examine the statistically significant pairwise correlations between three networks and define within-group correlations. The FNC differences between PTSD and non-PTSD groups were then assessed using a two-sample *t* test, as implemented in the FNC toolbox^43^.

### Cross Validation

Considering the limited sample size, a leave one out cross validation was performed to evaluate how reliable the findings of this study, if they have significant differences in within- and between-network connectivity^44^. In the cross validation, a SVM classifier with a radial basis function kernel was implemented by using the famous LIBSVM package^45^. After the mean Z values of each identified region and/or FNC values were extracted from each subject and normalized to [–1, 1], the classifier was trained by 19 datasets and tested by the left dataset, and the process repeated for 20 times. After classification, the results, namely the sensitivity, specificity, accuracy, and area under the curve (AUC) of the ROC, were calculated to evaluate the differentiation performance.
